# A novel graphene oxide-based ceramic composite as an efficient electrode for capacitive deionization

**DOI:** 10.1038/s41598-020-66700-8

**Published:** 2020-06-15

**Authors:** Khalil Abdelrazek Khalil, Nasser A. M. Barakat, Moaeed Motlak, Fahad S. Al-Mubaddel

**Affiliations:** 1 Department of Mechanical & Nuclear Engineering, College of Engineering, University of Sharjah, P.O. Box 27272, Sharjah, UAE; 2 Chemical Engineering Department, Minia University, El-Minia, 61519 Egypt; 3 Department of Physics, College of Science, University of Anbar, Anbar, Iraq; 4 Chemical Engineering Department, College of Engineering, King Saud University, Riyadh, 11421 Saudi Arabia; 5 King Abdallah City for Atomic and renewable Energy, Energy Research and Innovation Center (ERIC)In Riyadh, Riyadh, 11453 Saudi Arabia

**Keywords:** Engineering, Materials science

## Abstract

In this study, N-doped and TiO_2_-decorated graphene oxides were developed as efficient nonprecious electrodes for capacitive deionization. The activity of this new material was evaluated *in situ* and *in vivo*. The performance of the synthesized material was measured in different saline solutions (0.1, 0.5 and 1.0 M NaCl) as an electrolyte. The results showed that the new material exhibits very good performance (157 F/g at 5 mV/s and 1.0 M NaCl compared to 19.5 F/g for pure graphene oxide). In the desalination test, which was performed in batch mode, the salt adsorption capacity and the efficiency of salt removal were 9.2 mg/g and 98%, respectively. To check the stability, the desalination test was repeated several times, and no change in the performance was observed. The results provide evidence that the newly synthesized material is a potential electrode material for CDI water desalination with satisfactory salt removal ability.

## Introduction

Most existing desalination technologies require a great deal of energy. Even nuclear power has proven too expensive for desalination and is the most expensive water supply option. Thus, energy conservation has prompted researchers to develop new water desalination technologies^1,2^. Capacitive deionization (CDI) is a promising new technology compared to other traditional desalination methodologies, such as thermal or nonthermal distillation, due to its low energy consumption, low capital and processing costs, easy operation and maintenance processes, and environmentally friendly characteristics, with no hazardous chemicals used for electrode regeneration. Furthermore, CDI does not require high pressures, has low equipment and operational costs, employs low voltages and low power (low energy cost), is safe and involves no expendables. Small CDI units can be used in remote locations and run by solar panels, and some of the energy can be recovered by utilizing stored energy. CDI can achieve arsenic removal to below drinking water standards. CDI is strongly recommended as an environmentally friendly and economical technique for removing salt ions from saline water. The main concept of CDI is to use the electrosorption/desorption processes of an electric double layer capacitor as an electrochemical water purification method. By applying an external electrical potential (very low voltage, 1.3 V), salt ions in the feed water will be attracted by the oppositely charged electrodes. Reversing the potential leads to ejection of the desorbed ions and regeneration of the electrodes for a continuous desalination process^3^.

Researchers are in a race to develop CDI technology, and at the heart of this research are the electrodes. Effective electrodes should have high electrical conductivity, a high specific surface area with adjusted and well-controlled pore sizes, and excellent electrolyte wettability. The electrode materials should have a potential at zero charge, which is the potential at which anions and cations are adsorbed onto a surface (a voltage more negative than this potential will cause adsorption of cations, and a more positive voltage will cause adsorption of anions). Many innovative electrode materials have been registered in patents^4,5^. The most suitable materials that can be used as electrodes are carbon-based materials, including aerogels^6,7^, activated carbon (AC)^8^, mesoporous carbon (OMC)^9,10^, carbon nanofibers (CFs)^11^, carbon nanotubes (CNTs)^12^, and graphene^13^, which are the best candidates for CDI electrodes. Moreover, other high-performance carbon electrodes have been developed^7,14,15^.

The ability of an electrode to absorb ions basically depends on the surface properties of the electrode material, such as the electrical conductivity, surface area, and pore size and distribution. Therefore, carbonaceous mixtures, such as carbon nanotube and nanofiber (CNT–CNF) composite films, have also been proposed as electrosorption electrode materials^16^. Other electrode materials have also been investigated. Ryoo *et al*. used modified activated carbon cloth as an electrode to enhance the capacitive deionization performance. However, this was only very fundamental research^17^.

Among the reported nanostructure carbonaceous materials, graphene has drawn the most attention due to its very large surface area and excellent electrical conductivity^18–21^. Unfortunately, the known high hydrophobicity of graphene harms its performance in aqueous media due to the low contact angle, which adds additional resistance. However, the oxygenated groups on the surface of graphene oxide (GrO) make this material more hydrophilic than graphene. On the other hand, the electrical conductivity of GrO, compared to that of graphene, is negatively affected by these active groups. In fact, GrO’s electrical conductivity depends on the carbon/oxygen ratio in the sample. During the oxidation step, the sp2 carbons are replaced by sp3 carbons with oxygen functionalities, and this process will create a band gap by pulling the bands apart. Consequently, graphene oxide can behave as an insulator when graphene is fully oxidized. However, the partially oxidized GrO behaves as a semiconductor due to the appearance of sp3 and sp2 regions^22^, and the electrons can pass through the sp2 regions via Klein tunneling^22^. Insertion of a transition metal oxide and nonmetals within the GrO layers could play a crucial role in enhancing electron transfer^23^.

In this work, a fast and green synthesis route for controlling the morphological structure is presented. TiO_2_ and N-doped graphene oxides have been developed as efficient nonprecious electrodes for capacitive deionization. It is expected that this fabrication process will meet many CDI electrode requirements.

## Experimental

### Materials

Commercial graphite powder (<20 μm) purchased from Sigma Aldrich was utilized as a graphene precursor. Titanium chloride solution (Conc 20% TiCl_4_ from Cica-reagent, Japan) and urea hydrazine were used without any further purification.

### Preparation

The graphene oxide (GrO) was synthesized via a modified Hummer’s method^24^. More details regarding preparation of GrO can be found in our previous paper^25^. Then 0.3 g of the prepared graphene oxide were ultra-sonicated for 40 min in 200 ml water. Later on, 150 ml titanium chloride solution (Conc 20%), 0.1 g urea (as a source of nitrogen) and 150 micro liter hydrazine were added. All these mixtures were subjected to closed reflux system at 150 °C for 5 h. Then filtration and drying at 60 °C under vacuum for 1 day.

### Characerization

X-ray diffraction was conducted to analyse the phase and crystallinity using Rigaku X-ray diffractometer (Rigaku Co., Japan) with Cu Kα (λ = 1.54056 Å). 2θ angles from 10° to 80° has been used. A spectrometer (JY H800UV) equipped with an optical microscope was used to collect Raman spectra at room temperature. Scanning electron microscope (SEM, JEOL JSM-5900, JEOL Ltd., Japan), field-emission scanning electron microscope (FE-SEM, Hitachi S-7400, Japan) and Transmission electron microscope (TEM, JEOL JEM-2010, Japan) operated at 200 kV equipped with EDX analysis has been used to investigate the surface morphology of the produced samples.

CDI is a solt seperation process that can be simply described as adsorption/desorption processes occurring on the electrode surface by means of electrostatic force. The salt ions are adsorbed on the charged electrode and form an electric double layer (EDL). As polarity reversed, the regeneration process take place and the ions are desorbed from the electrode surface and released back to the solution. The electrode is the main part of CDI unit. the salt adsorption capacity (SAC) of the CDI electrode material strongly depends on the properties including high electrical conductivity, large capacitance, high wettability and electrochemical stability. The salt adsorption capacity was analyzed by cyclic voltammetry cell simulate the CDI flow cell using the proposed materials. Cyclic voltammetry measurement was done by using different NaCl concentrations (0.1, 0.5 and 1 M) after adjusting the sweep potential range from −0.4 to 0.6 V vs Ag/AgCl in the electrochemical cell. This range was selected according to that reported for graphene and graphene doped materials^26,27^. Three-electrode system were used: counter electrode from platinum wire, Ag/AgCl, reference electrode and the prepared materials as the working electrode. VersaStat4 potentiostat device was used to control the whole system. The specific capacity was simply calculated by integrating the area under the CV curve to determine the average value according to the following equation^28,29^.

1 $${\rm{Cs}}=\frac{{\int }^{}IdV}{2vVm}$$

where Cs is the specific capacitance (F/g), I is the current (A), ν is the scan rate (V/s), ΔV is the applied potential window (V), and m is the mass of active electrode material (g). pH meter was utilized to track the pH during this study. The pH was around 6. Although it is well known that the carbonaceous materials are featured by high chemical stability, in CDI a slight dissolution occurs which can be detected by a decrease in the pH value of the effluent due to release of the hydrogen ions. Interestingly, when utilizing the proposed material electrode, there is almost no change in the pH of the effluent which concludes higher stability.

## Results and discussion

### Electrode characterization

#### Morphology

The Hummers approach was adopted to synthesize single- or few-layered chemically exfoliated graphene oxide (GrO), followed by postprocessing with thermal treatment or chemical reduction to achieve a better reductive degree of graphene, also known as reduced graphene oxide (rGO). Moreover, GrO with enough oxygen functional groups, small particles, and massive exposed edge sites offers a promising matrix for surface modification, especially for heteroatom doping. TiO_2_ is one of the most widely used semiconductors with supercapacitor electrodes that exhibit excellent performance and a wide band gap and are placed in contact with a redox electrolyte. The purpose of using TiO_2_ and N-doped GrO is to improve the electrochemical characteristics, as TiO_2_ intercalates between the graphene oxide nanosheets.

**Figure 1:**
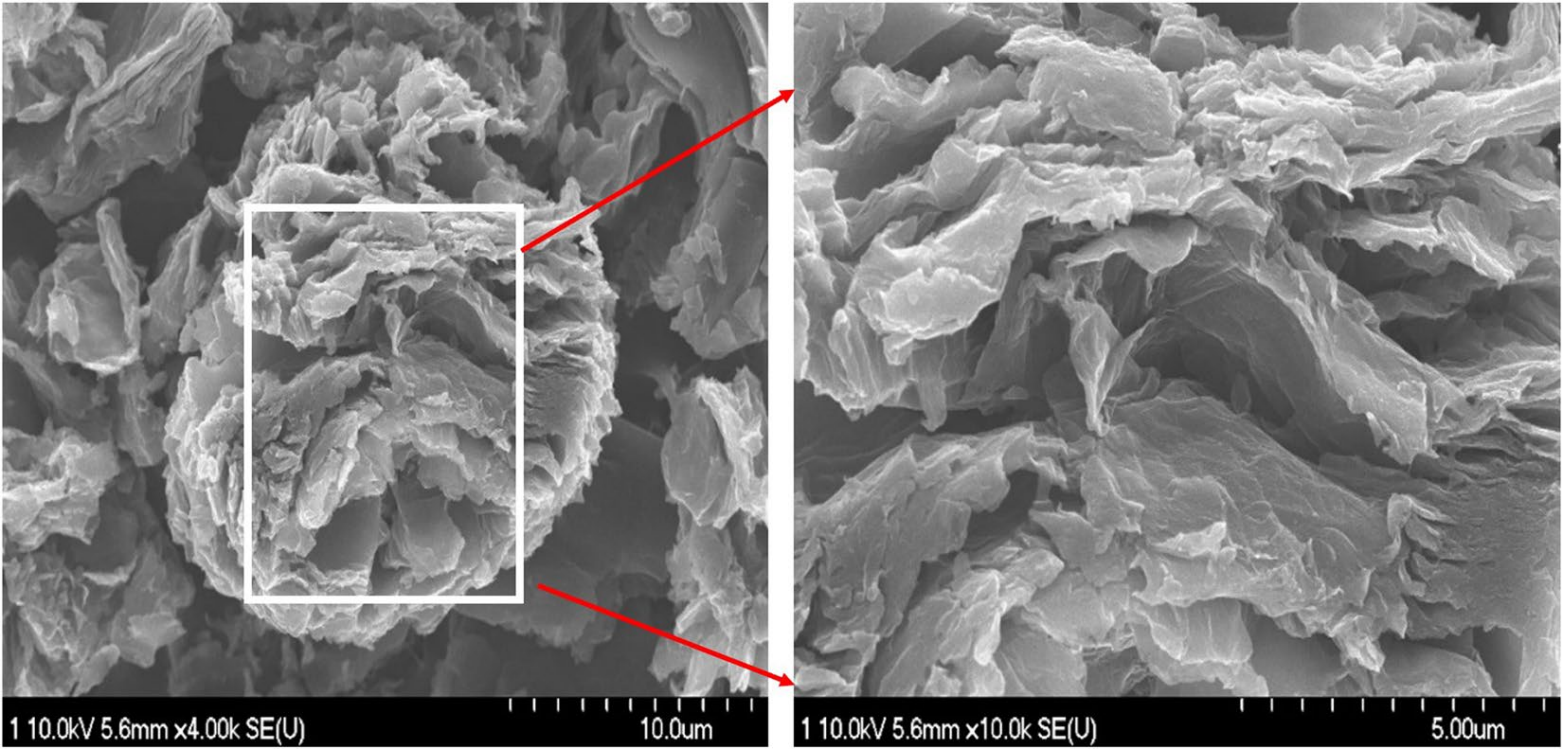
SEM images of the prepared materials showing the formation of wrinkled and layered flakes-like structures.

The morphology of the TiO_2_ and N-doped graphene oxide nanostructure can be easily controlled, as shown in Fig. 1. The left image shows the TiO_2_ and N-doped GrO semisphere with a diameter of nearly 10 µm. This SEM image confirms that the sample contains spherical TiO_2_ nanoparticles. The right image shows a magnified part of the image on the left. TiO_2_ and N-doped GrO is in the form of layers with a thickness on the nanoscale. These introduced forms of TiO_2_ and N-doped GrO separating layers are very effective for use in capacitive deionization with highly improved salt adsorption capacity; consequently, an optimum CDI electrode can be obtained.

#### Internal structure

**Figure 2:**
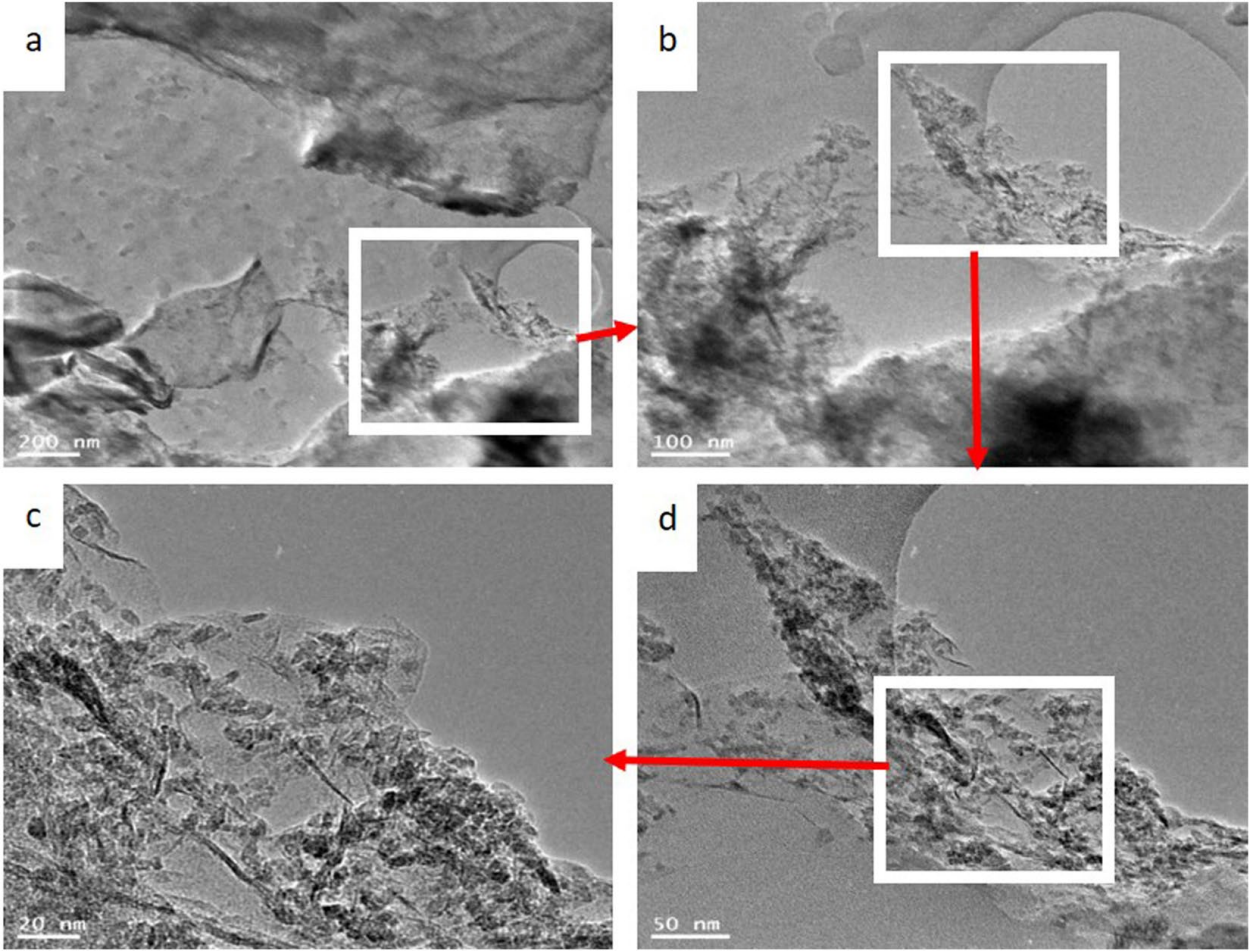
Low- and high- magnification TEM micrographs.

The TEM images presented in Fig. 2 clearly show the ultrathin needle-like structure of the TiO_2_ and N-doped GrO material. Ultrathin wrinkled graphene oxide NPs with systematic distribution can also be observed. The image indicates that TiO_2_ intercalated into the flexible and ultrathin wrinkled graphene oxide sheets with a few layers to form a needle-like structure. Decoration of the prepared graphene oxide by small particles of TiO_2_ could be proved by TEM images (Fig. 2C,D), and more evidence for the attachment of TiO_2_ nanoparticles onto the carbonaceous support was also provided by XPS analysis.

#### Chemical composition

**Figure 3:**
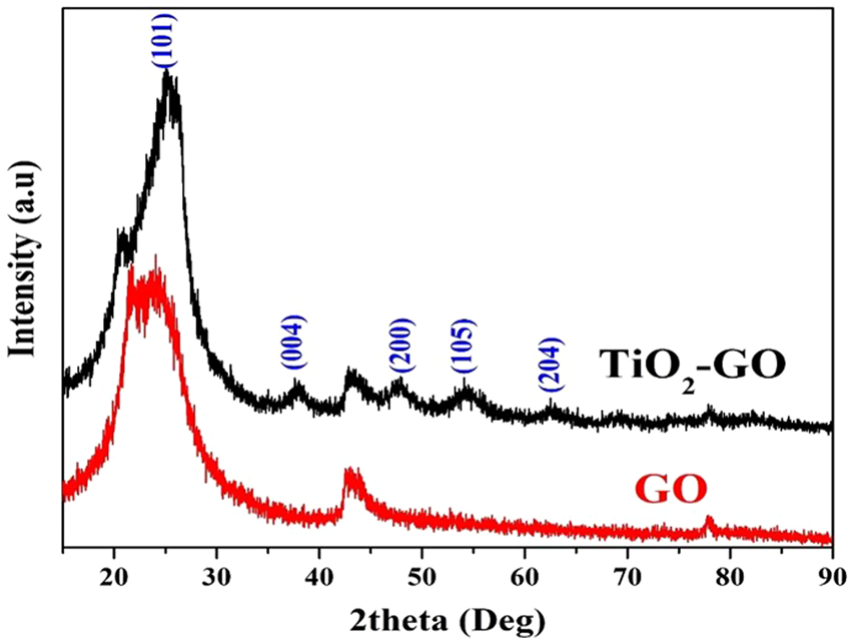
XRD patterns for the pristine and TiO_2_-decorated & N-doped GO. The crystal plans of the TiO_2_ (anatase) phase.

Figure 3 demonstrates the XRD patterns of the synthesized composite and pristine graphene oxide. The observed broad diffraction peak at 2θ angles of 22.2–26.8° in the case of bare graphene oxide confirms the formation of graphene oxides and simultaneously indicates disordered stacking. However, three distinctive additional diffraction peaks at 2*θ* values of 25.3°, 37.8°, 53.9° and 62.6° corresponding to the (101), (004), (200) and (204) crystal planes, respectively, indicate the formation of the anatase phase of TiO_2_.

**Figure 4:**
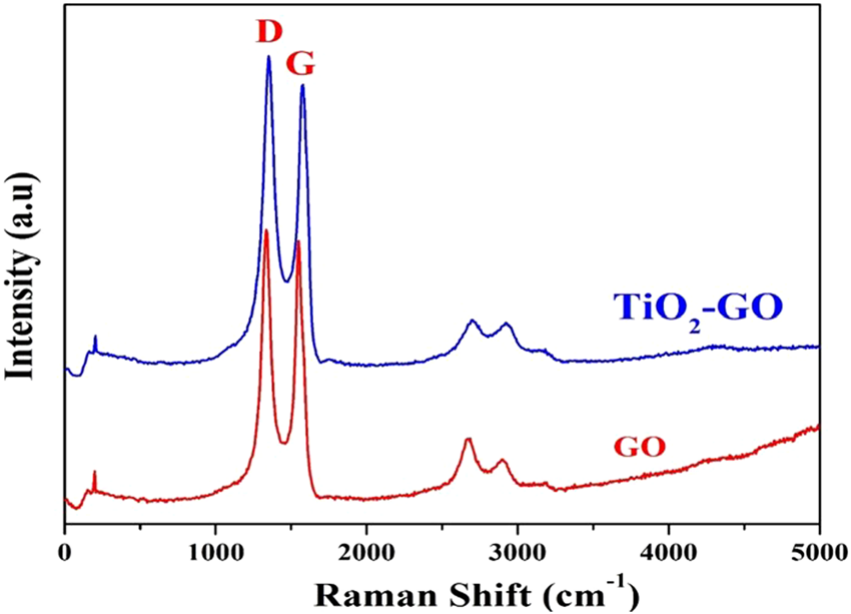
Raman spectroscopy for the pristine and TiO_2_-decorated & N-doped GO. The data indicate that decoration of GO by TiO_2_ nanoparticles and doping by nitrogen did affect the GO structure.

The rotational, vibrational and other low-frequency modes of compounds can be easily investigated by Raman spectroscopy^30^. This technique can efficiently and easily prove the formation of graphene oxide, as it can distinguish GrO from the utilized graphite precursor. For instance, in the case of pristine graphite, the obtained Raman spectrum shows a prominent G peak at 1581 cm^−1^, which corresponds to the first-order scattering of the E2g mode^31^. However, the G band is broadened and shifted to 1594 cm^−1^ in the case of graphene oxide. Moreover, because of extensive oxidation, the D band, which appears at 1363 cm^−1^, becomes dominant, indicating that the in-plane sp2 domains decrease. Finally, the D/G intensity ratio in the case of graphene oxide approaches unity, while that of graphite is very small^31^. Figure 4 displays the Raman spectra of the prepared graphene oxide in pristine form and after decoration by TiO_2_ nanoparticles and doping with nitrogen. It can be easily concluded from these spectrum corresponding to pristine GO that the chemical procedure utilized was successfully applied to prepare graphene oxide. Raman spectroscopy can also easily distinguish the two compounds. For the reduced graphene oxide, the intensity of the D band is higher than that of the G band. Furthermore, the D/G ratio is very high, especially in the case of single (or few)-layered graphene. However, in our case, as shown in the figure, no change in the ratio can be observed after decoration and doping. Actually, the presence of functional groups on the surface of graphene oxide makes it preferable in capacitive deionization applications due to the enhanced ability for ion adsorption compared to graphene.

**Figure 5:**
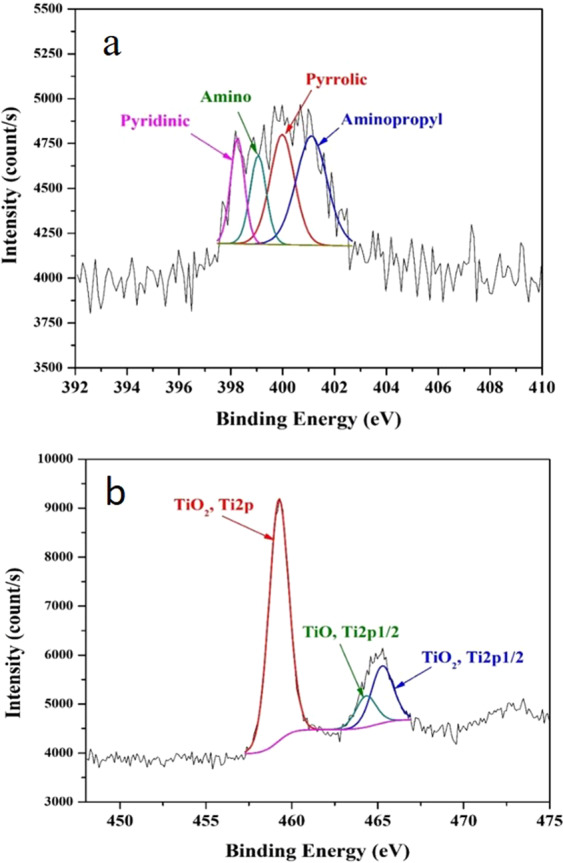
XPS spectra for the prepared composite within the binding energy range of N1s; (**A**), and Ti2p; (**B**).

Urea was added during the hydrothermal treatment to incorporate nitrogen atoms within the GO sheets. To verify the presence of nitrogen as a doping element in GO, XPS analysis was used. X-ray photoelectron spectroscopy (XPS) analysis is a powerful technique to investigate surface elements and their electronic structure. Moreover, some elements cannot be detected by XRD, such as nonmetals and even noncrystalline metals. However, XPS is a powerful analytical technique that can detect all elements except hydrogen and helium. Figure 5A displays the scanning results at the binding energy range corresponding to the N1s orbital. As shown in the figure, a good amount of nitrogen in the form of pyridinic (approximately 398 eV), amino (approximately 399.05 eV), pyrrolic (approximately 399.63 eV), and aminopropyl states could be detected^32^. This result confirms the doping of the prepared composite by nitrogen. Decoration of the prepared graphene oxide by small particles of TiO_2_ could be proven by XRD analysis (Fig. 3) and TEM images (Fig. 2), and more evidence for the attachment of TiO_2_ nanoparticles to the carbonaceous support could also be provided by XPS analysis (Fig. 5B). As shown in the figure, titanium was observed to have different electronic structures: Ti2p at a binding energy of 458.7 eV^33^ and Ti2p1/2 at 465.2^34^. The peak observed at a binding energy of 464.2 can be assigned to the Ti2p1/2 electron in the TiO compound^35^.

### Electrochemical measurements

Cyclic voltammetry (CV) is a good electrochemical technique to investigate the surface activity of functional materials.

**Figure 6:**
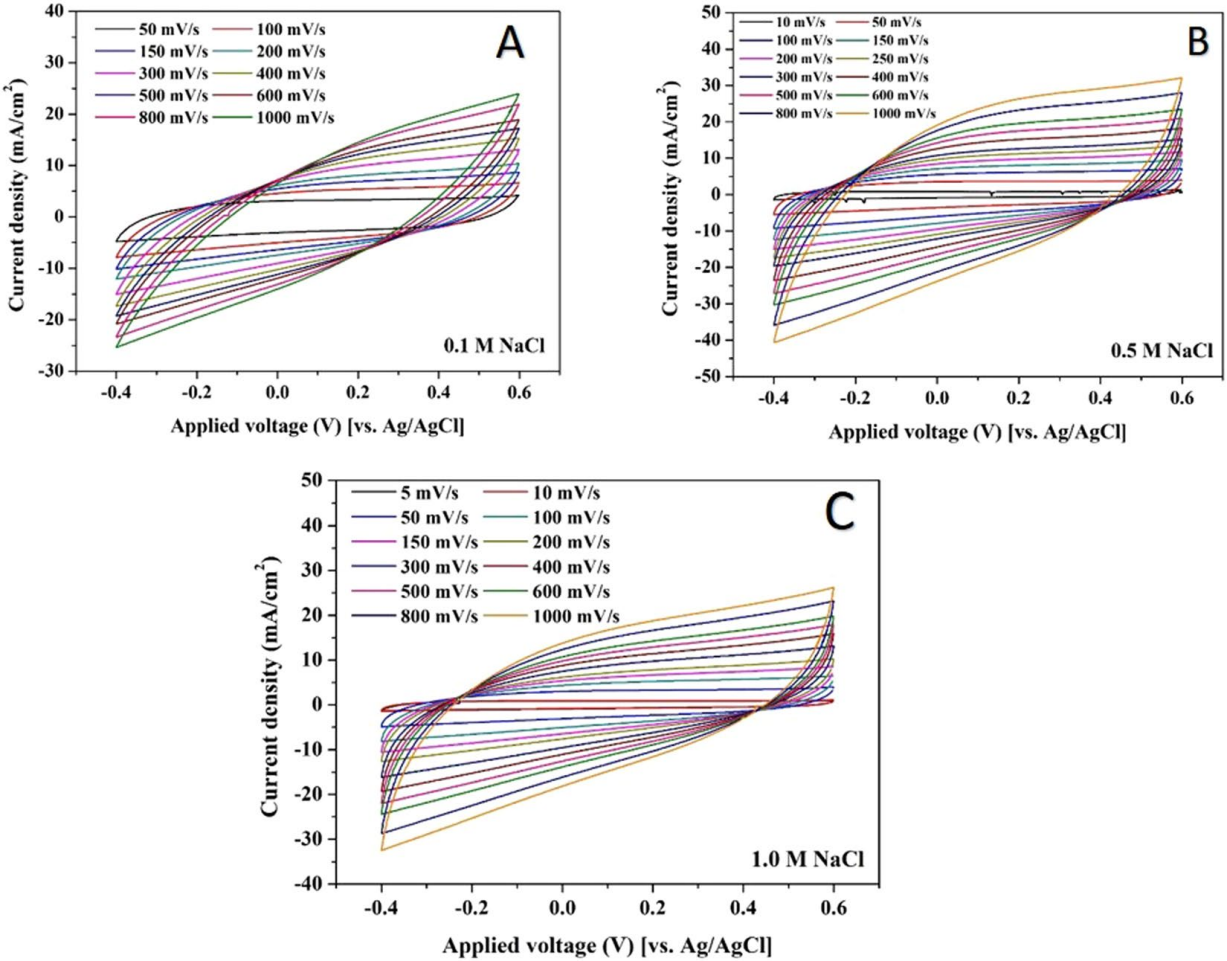
Effect of scan rate change on the cyclic voltammetry measurement in case of TiO_2_-N-GrO composite using a salt solution concentration of 0.1, 0.5 and 1.0 M NaCl at 25 °C.

Figure 6 displays the CV results for the prepared TiO_2_ and N-doped GrO electrode at different scan rates. The analyses were conducted using different NaCl solution concentrations (0.1, 0.5 and 1.0 M) at 25 °C in an applied voltage window of −0.4~0.6 V (vs. Ag/AgCl reference electrode). As shown in the figure, smooth ellipse-shaped cycles were obtained at all the assigned scan rates for all concentrations. This finding indicates, due to the absence of any redox peaks, that the surface is very stable and free of any functional groups. Moreover, an increase in the cycle width indicates an increase in the amount of adsorbed ions, which can be understood as an improvement in the formation of the electric double layer. Actually, the high surface area due to the exfoliated GrO sheets increased the number of absorbed ions. Additionally, no chemical reactions are involved, allowing the ultracapacitor to be rapidly charged and discharged (i.e., adsorption and desorption cycles while serving as an electrode in the CDI unit) thousands of times, which is highly beneficial in CDI technology.

**Figure 7:**
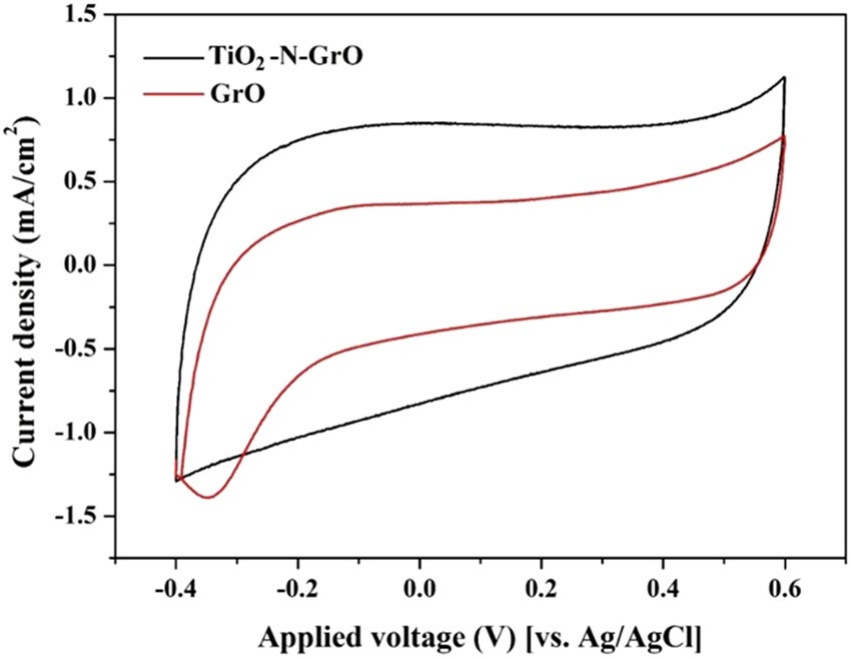
Influence of the proposed doping by TiO2 and nitrogen on the cyclic voltagrammograms of the graphene oxide (GrO) at 5 mV/s and 1.0 M NaCl.

Notably, our previous study concluded that utilizing TiO_2_ only as a dopant did not distinctly enhance the electrochemical absorption of the ions, especially at a content close to that used in this study^25^. To show the advantage of the utilized codoping strategy (TiO_2_ and nitrogen), Fig. 7 displays the CV measurements for the pristine and TiO_2_ and N-doped GrO in the presence of 1.0 M NaCl solution at a scan rate of 5 mV/s. As shown in the figure, a very considerable enhancement in ion adsorption for the introduced material was obtained compared to that of pristine graphene oxide. Moreover, the observed tail (at the negative potential) in the case of GrO can be assigned to the oxygen evolution reaction. In other words, pristine GrO possesses electrocatalytic activity toward the water reduction reaction to produce oxygen gas. Even though this activity is very small, it is undesirable in CDI technology. In addition to the improvement in ion adsorption, the proposed doping process annihilates the electrocatalytic activity.

**Figure 8:**
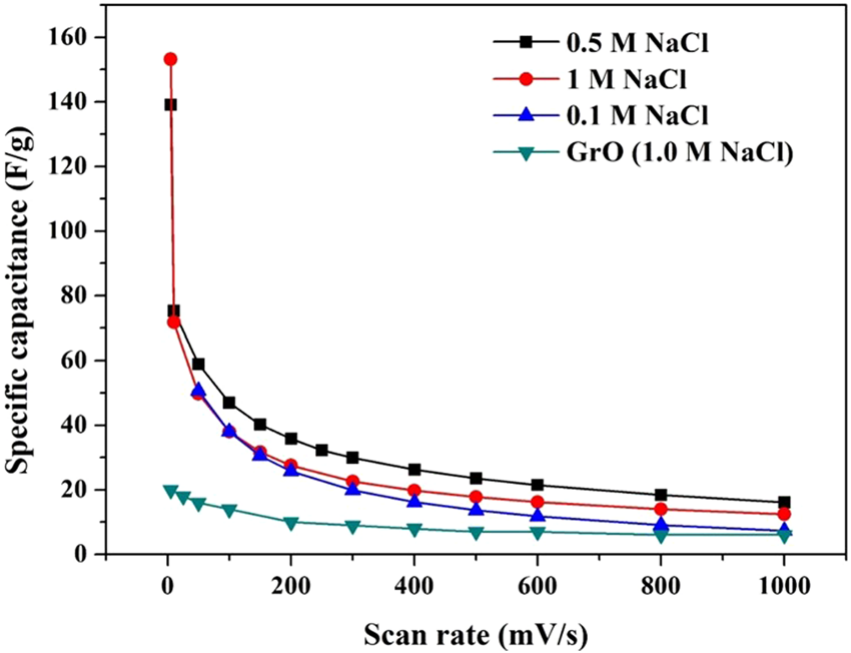
Specific capacitance values at different scan rates for the pristine graphene oxide (GrO), and the proposed TiO2-N-GrO composite.

Numerically, the observed cycles in the above figures can be translated into the specific capacitance values by applying Eq. 1 for calculations. Figure 8 demonstrates the estimated specific capacitance values at different scan rates. As shown, at all the utilized saline solution concentrations, the specific capacitance is high. Compared to the pristine GrO, in 1.0 M NaCl, the prepared catalyst reveals very good performance. Numerically, the specific capacitance at 0.005 V/s is 157 and 19.5 F/g for TiO_2_ and N-doped GrO and pristine GrO, respectively, which translates to an 8-fold increase in the specific capacitance upon doping of graphene oxide.

**Figure 9:**
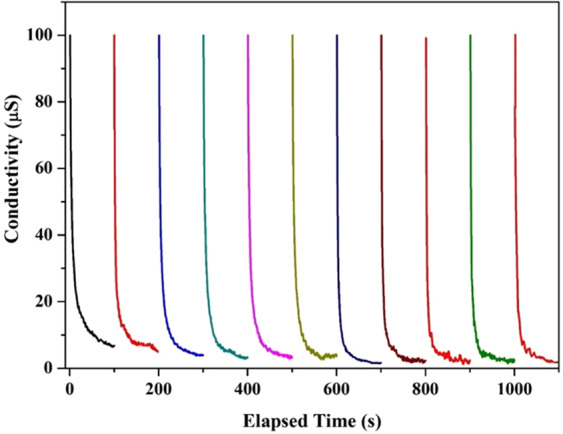
Multiple cycles for the desalination performance of the proposed TiO2-N-GrO in a batch mode at 0.4 V (vs. Ag/AgCl) and 25 oC. 15 ml NaCl solution was used.

**Figure 10:**
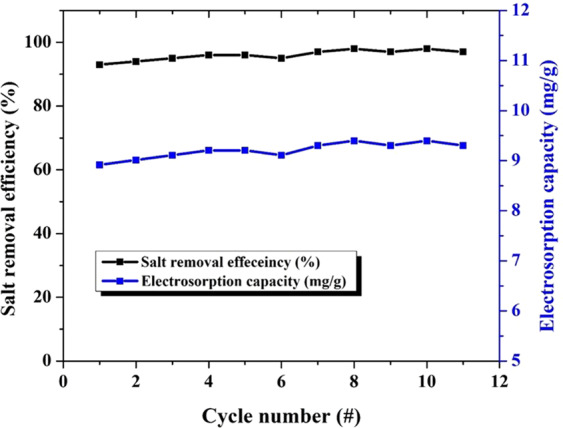
salt adsorption capacity and salt removal efficiency for the proposed electrode at different cycles.

Practically, the real performance of any proposed electrode for the CDI unit is estimated by investigation of its ion adsorption and durability. Figure 9 shows the ion adsorption behavior on the surface of the proposed electrode until the saturation state for several cycles. As shown in the figure, for all cycles, saturation of the proposed electrode requires approximately 100 s. Interestingly, the performance of the electrode was not affected by utilization for many cycles, as shown in the figure. This finding can be attributed to the absence of any functional group on the surface, as concluded from the CV measurements. The salt adsorption capacity (SAC) and the salt removal efficiency (${\eta }_{d}$) of the CDI electrode could be determined by the salt concentration change during the adsorption process^36,37^:

2 $${\eta }_{d}=1-\left(\frac{{C}_{{\rm{eff}}}}{C{\rm{o}}}\right)\times 100$$

3 $$SAC=\frac{(C{\rm{o}}-{C}_{{\rm{av}}})V}{M}$$

where ${C}_{{\rm{av}}}$ is the average NaCl concentration of the effluent during the adsorption process, $C{\rm{o}}$ (mg/L) is the initial NaCl concentration*, V* (L) is the total volume of the solution, and *M* (g) represents the mass of the electrodes. Figure 10 demonstrates the salt removal efficiency and the salt adsorption capacity of the utilized electrode for different cycles. As shown in the figure, the salt removal efficiency of the desalination process over the surface of the prepared electrode is excellent, with a value of approximately 98%. Moreover, the average salt adsorption capacity is approximately 9.2 mg/g. Furthermore, both the salt removal efficiency and the salt adsorption capacity are almost constant within the utilized cycles, which indicates the very good stability of the proposed material. Overall, the proposed electrode fulfils many requirements for an optimum electrode material for a capacitive deionization unit.

## Conclusion

The Hummers method is a good chemical procedure for the preparation of graphene oxide nanosheets through a chemical route. Hydrothermal treatment of the prepared graphene oxide in the presence of titanium chloride and urea results in decoration of the carbonaceous sheets by TiO_2_ nanoparticles and doping by nitrogen atoms. Incorporation of the metal oxide and doping by nitrogen distinctly improve the specific capacitance of the graphene oxide, resulting in an 8-fold increase in the specific capacitance. As an electrode in the capacitive deionization unit, the modified graphene oxide shows high salt removal efficiency and satisfactory salt adsorption capacity, with high stability for both parameters. Overall, this study opens a new avenue for titanium oxide nanoparticles and nitrogen as effective dopants to improve the characteristics of carbonaceous materials to be exploited as efficient electrodes in capacitive deionization units.
